# Vitamin requirements during stem cell transplantation: a systematic review

**DOI:** 10.1007/s00520-022-07409-4

**Published:** 2022-11-09

**Authors:** Bronwyn Segon, Leroy Lam, Hei Yan Chan, Sarah Andersen, Teresa Brown, D’Arcy Kenway, Judy Bauer

**Affiliations:** 1 The University of Queensland, Brisbane, QLD Australia; 2 The Royal Brisbane and Women’s Hospital, Brisbane, QLD Australia; 3 Monash University, Melbourne, VIC Australia

**Keywords:** Vitamin deficiency, Stem cell transplant, Vitamin supplementation, Malnutrition, Systematic review

## Abstract

Patients undergoing stem cell transplantation (SCT) are at high risk of malnutrition during the acute post-transplantation period. This systematic review aimed to collate and analyse the evidence for vitamin requirements post-SCT. A systematic search of five databases was conducted to include studies published until March 2021. The review utilised the Preferred Reporting Items for Systematic Reviews and Meta-analyses (PRISMA) framework. Inclusion criteria consisted of adults undergoing SCT who received vitamin supplementation or had their vitamin levels monitored up to 100 days post-SCT. Studies with paediatric patients or those that looked at vitamin derivates such as folinic acid were excluded. Main outcomes included vitamin deficiency and relevant clinical outcomes. Eleven studies (*n* = 11) were eligible for inclusion with five rated as neutral quality and six as positive quality. Five studies focused on allogenic SCT, two on autologous SCT and the remaining included a mix of both. Eight studies monitored vitamins levels post-SCT, and seven studies provided vitamin supplementation. Three studies (one provided supplementation) found a high prevalence of vitamin D deficiency (23–60%) prior to SCT. Findings indicate an unclear association between vitamin deficiency and post-SCT complications including acute graft-versus-host-disease, oral mucositis, and mortality. The GRADE certainty of evidence across these outcomes was low or very low. It is unclear if supplementation is needed during SCT, though assessing vitamin D levels prior to transplant should be considered. Further large observational studies or randomised control trials are required to establish vitamin requirements and guide supplementation protocols during SCT.

## Introduction

A Stem Cell Transplant (SCT) is used to effectively treat several non-malignant and malignant disorders. With advances in the allogeneic stem cell transplantation procedure (allo-SCT) [1] and the rise of autologous SCT [2], it is now globally estimated that more than 50,000 individuals undergo SCT annually [3]. Improvements in donor selection, conditioning regimes and supportive therapy have resulted in superior transplant outcomes and increased survivorship [1]. Although SCT can be a life-saving procedure, the risks of severe morbidity and mortality are significant [1, 4, 5]. Transplant outcomes are impacted by disease stage, diagnosis, stem cell source, age, prior treatment regimens and nutritional status [6].

The 100 days following SCT is a crucial window with the events and complications that occur within this acute post-transplant period being strong indicators for the success of the procedure and predictors of long-term survivorship [7, 8]. Acute complications can include toxicity, acute graft-versus-host-disease (aGvHD) and aplasia [3]. The nutritional status of transplant recipients during this acute phase is extremely important as malnourished patients have an increased risk of post-transplant toxicity, aGvHD, longer hospitalisation and have lower survival rates [9–14]. Patients undergoing SCT may have increased energy requirements and are extremely susceptible to becoming malnourished [15–17]. These possible increased requirements may be related to the disease itself, catabolic effects of the conditioning regimen, presence of infection and treatment-related toxicities [6, 18]. Poor nutritional status is often further exacerbated by inadequate oral intake with nutritional support frequently required. As such, it is recommended that all patients undergoing SCT receive routine malnutrition screening and that nutrition support therapy is used for patients who are malnourished or who are unable to ingest/absorb nutrients for a prolonged period [3, 19, 20]. The European Society for Parenteral and Enteral Nutrition (ESPEN) recommends enteral nutrition (EN) as opposed to parenteral nutrition (PN), ‘unless the use of EN is contraindicated by severe mucositis, frequent vomiting, ileus, severe malabsorption, protracted diarrhea or symptomatic GvHD’ [20].

A decrease in micronutrient intake is likely to occur in conjunction with decreased oral intake post SCT [3, 11, 21, 22]. Currently, there is only one set of guidelines addressing the vitamin requirements in patients undergoing SCT — The Brazilian Nutritional Consensus in Hematopoietic Stem Cell Transplantation: Adults 2020 — which recommends that electrolytes, minerals, vitamins, and trace elements supplementation are adjusted to individual needs [3]. However, the micronutrient requirements of an individual may vary due to the presence of ‘GVHD, antibiotics, metabolic stress, immunosuppressants, diarrhea and vomiting’ (3 p. 35). Despite this, the guidelines only provide general recommendations of daily amounts of electrolytes, vitamins, and trace elements for patients on PN therapy and recommend daily electrolyte monitoring [3].

The emphasis on nutrition support to maintain health status is well justified and supported by the literature. However, routine vitamin and trace element monitoring is not common [3]. As such, there is a need to synthesize and assess the current evidence regarding the vitamin requirements for SCT patients to better inform current practice. This review aims to determine (1) vitamin requirements and if supplementation is required during stem cell transplantation or in the acute post-transplant period (up to 100 days) and (2) association between vitamin status and risk of post-transplant oral mucositis (OM), aGvHD and mortality.

## Methods

This systematic review utilised the Preferred Reporting Items for Systematic Reviews and Meta-Analyses (PRISMA) 2020 statement [23] and was registered on the 19^th^ of March 2021 (243,773; https://www.crd.york.ac.uk/PROSPERO).

### Eligibility criteria

Included studies consisted of adult patients who were undergoing allogeneic or autologous stem cell transplantation (SCT). Eligibility criteria included studies where vitamin supplements were provided and/or when vitamin levels were monitored prior to SCT and up to 100 days post SCT. Outcomes included vitamin levels, prevalence of vitamin deficiency, and other relevant SCT clinical outcomes, including GVHD, mortality and survival rate, oral mucositis, variation in quality of life, relapse rate and prevalence of deficiency related health issues. Studies with participants aged < 18 years, initial monitoring more than 100 days post-transplant and studies that included only participants with aGvHD or chronic GvHD (cGvHD) were excluded. Studies that included pediatric participants were excluded due to the differing nutritional needs [24]. Studies that focused solely on trace element levels, or those that provided high protein and energy supplements, glutamine, fish oil, probiotics, or prebiotics, with no provision of vitamin supplements or monitoring for deficiency were also excluded. Of note, studies that included vitamin derivates, for example B9 derivates such as folinic acid or leucovorin, were not included. Additionally, review articles, case studies, conference abstracts, letters to the editor and case series were excluded. Lastly, studies were excluded if the full text was not available.

### Search strategy

A literature search was conducted in PubMed, CINAHL, Embase, Scopus and Cochrane Library to include English human studies published from inception until March 2021. Reference lists of included studies were checked for additional or missing relevant studies. The search included the terms ‘bone marrow transplant’, ‘bone marrow graft’, ‘stem cell transplant’, ‘haematopoietic cell transplant’, ‘SCT’, ‘vitamin’, ‘multivitamin’, ‘provitamin’, ‘supplement’, ‘deficiency’, ‘requirement’, ‘level’, ‘intake’, ‘diet supplement’, ‘food supplement’, ‘dietary supplement’, ‘nutritional supplement’, ‘nutrition supplement’, ‘nutraceutical’, ‘micronutrient’. The full search strategy that was used for Embase is outlined in Appendix.

#### Data extraction

The titles and abstracts of each study were first reviewed by two authors independently against the inclusion criteria. Studies which met the inclusion criteria were then read in full, again by two authors, and those which still met the inclusion criteria were deemed eligible for risk of bias analysis and data extraction. Disagreements regarding whether a study should be included or the reason for exclusion were resolved through discussion until a consensus was reached with the assistance of a third author. Data extracted from eligible studies included participant’s demographics (sample size, age, gender, diagnosis, type of donor, transplant type), study inclusion and exclusion criteria, study design, definition of micronutrient consumption, prevalence of vitamin deficiency and any relevant clinical outcomes, including post-transplant complications (e.g., GVHD, infections, mortality). Authors of included studies were contacted if missing data or additional information was required. Two authors were involved in data extraction of included studies. Disagreements regarding extracted data were resolved through discussion until an agreement could be reached.

#### Data synthesis and interpretation

The Grading of Recommendations, Assessment, Development and Evaluation (GRADE) system was used to assess the certainty of the body of evidence for outcomes OM, aGvHD and mortality [25]. Assessment was carried out by two independent authors and reviewed by a third author if any discrepancies occurred. A narrative synthesis of findings was completed with each outcome assessed for bias, inconsistency, indirectness, imprecision, and publication bias.

## Results

**Fig. 1:**
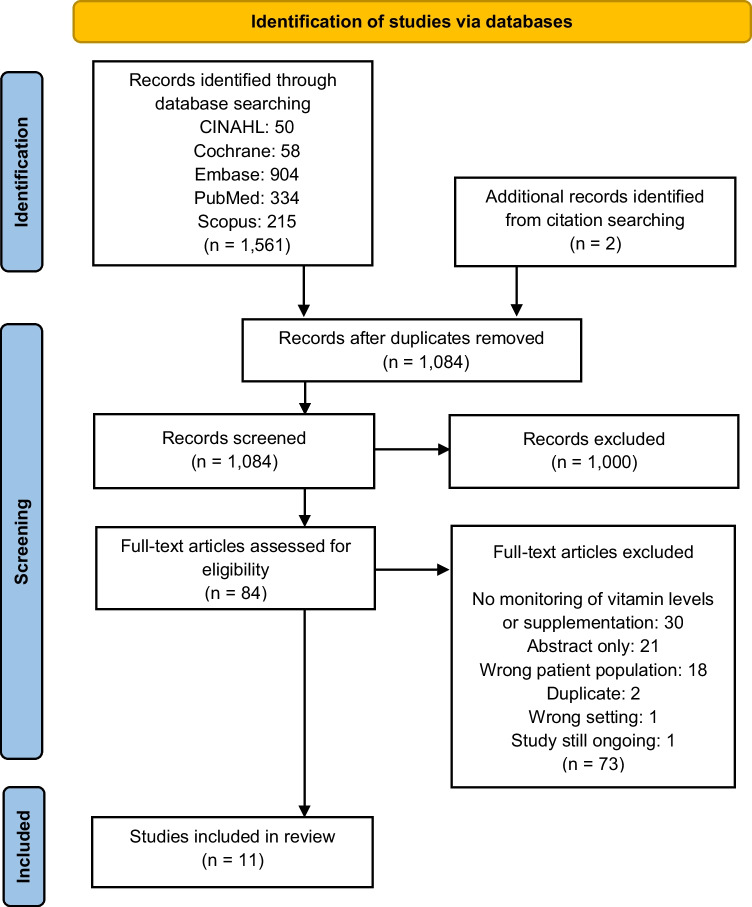
PRISMA flowchart

In the literature search, 1082 studies were identified for screening with two studies added after reviewing the included articles’ citations and reference lists. Overall, 11 studies met the inclusion criteria (Fig. 1). Two studies were randomised controlled trials with the rest being observational cohort studies. Two of the observational cohort studies used a retrospective design utilising medical records whilst a prospective design was used in seven of the studies. Study population consisted of patients undergoing SCT where they had been diagnosed with a haematological malignancy. Sample size varied from 15 to 311 participants. Study locations included the USA (*n* = 5), New Zealand (*n* = 1), Denmark (*n* = 1), Japan (*n* = 1), Germany (*n* = 1), Switzerland (*n* = 1) and Iran (*n* = 1) (Table 1).

**Table 1:** Study demographics with key findings

Study, study location and study design	Sample size [n, gender], age (mean), and transplant type	Feeding route	Vitamin of interest, supplemen-tation observed *(Y/N)*	Definition of supplementation	Frequency of micronutrient monitoring	Main outcomes measured				Other findings
						GVHD	Mortality and survival	Oral mucositis	Deficiency	
High et al. (2002), USA, PCS [26]	*n* = 120 (35% male) Age: 46 (9.7) TT: 24% Allo: 19% Auto: 81%	PN was provided when patients were not able to meet their EER orally	Vit A, Vit E ***(Y)***	Daily multivitamins given orally or through PN (based on USA dietary allowance for vitamins). Specific amount and duration not described	Plasma concentrations on baseline, Days 0, + 7, + 14 post SCT	-	Neither plasma retinol nor a-tocopherol was associated with overall mortality	The severity of mucositis was a strong predictor of low plasma retinol on Day 7 (*p* = 0.001)	82 of 120 (68%) of the patient group had hyporetinolemia (≤ 1.05 mol/L) and 37 (31%) had at least one plasma concentration ≤ 0.70 mol/L (retinol deficiency) between baseline to Day + 14 All plasma tocopherol levels were within normal limits	Low plasma retinol was associated with an increased risk of herpes zoster infection (95% CI: 0.4, 3.9) Plasma α-tocopherol was not associated with any clinical event measured
Robien et al. (2006), USA, RCS [27]	*n* = 311 (59% male) Age: 40.5 (9.1) Allo: 100%	97% of cohort received PN as the primary source of nutrients	Vit B9 ***(Y)***	85% of cohort had folic acid intake from parenteral solutions (3 times weekly) and daily oral multivitamin supplements (1 mg) between Day 0 and + 18 Oral supplements were used when patients able to absorb > 50% nutrients enterally	Levels not monitored with cohort divided according to averaged folic acid intake (≤ or > 400 μg/day) from patient records between Day 0 and + 18	Folic acid intake per day had no statistically significant association with risk of aGVHD	Folic acid intake per day had no statistically significant association with survival	No statistically significant association with OMI scores when comparing patients’ folic acid intake	-	Concurrent folic acid supplementation did not impair MTX effectiveness or toxicity in the cohort
Glotzbecker et al. (2013), USA, RCS [28]	*n* = 53 (58% male) Age: Median 44 (19–57)** Allo: 100%	Not described	Vit D ***(Y)***	13 patients received a standard multivitamin pre SCT. Specific amount of vit D in multivitamin not described 5 patients received Vit D supplementation (200–400 IU) combined with Ca^2+^ supplements pre SCT Duration not described	Serum samples obtained pre SCT	No significant associations were observed in cumulative incidence of grade II–IV aGvHD (53.1% in lower Vit D group vs 33.3% in higher Vit D group, *p* = 0.13) The CI of cGVHD was 63.8% in the lower Vit D group versus 23.8% in higher Vit D group (*p* = 0.009)	PFS and OS were similar between the cohort groups (51% in lower Vit D group vs 47% in higher Vit D group, *p* = 0.61; 53% in lower Vit D group vs 50% in higher Vit D group, *p* = 0.57)	-	60% of the patient group were classed in the deficient range (< 25 ng/mL) on baseline	-
Jonas et al., (2000), USA, RCT [29]	*n* = 24 (54% male) Patient subgroup *n* = 12 Age: Mod-PN: 38 (3)** *CON*: 41 (3)** Allo: 38% Auto: 62%	Patients received either standard PN or a modified PN containing only micronutrients and a small amount of lipid after transplant. PN discontinued when oral intake > 50% of EER for 3 consecutive days. Duration not described	Vit C, Vit E, ***(Y)***	Both standard PN and mod-PN formulas had: Vit C: 200 mg in vitamin prep + additional 500 mg daily Vit E: 20 mg in vitamin prep	Blood was obtained on baseline, Days + 1, 3, 7, 10, and 14 post-transplant	-	-	-	All monitored micronutrients were within lower normal range throughout the study	Plasma α- and γ-tocopherol levels decreased significantly from baseline to Day 14 (19.8 vs 15.3 μmol/L, *p* < 0.05; 5 vs 3 μmol/L, *p* < 0.001 respectively), but α-tocopherol value remained in the low-normal range (12–42 μmol/L). Normal range of γ-tocopherol not described Vit C levels increased from the lower end of normal ranges (23–125 μmol/L) on baseline to the midpoint of the normal range during PN administration
Gjaerde et al. (2021), DNK, PCS [30]	*N* = 115 (51% male) Age: 50*** (18–71) Allo: 100%	Patients had received PN, but formulation and duration not described	Vit E ***(N)***	-	Plasma α-tocopherol concentration on baseline and Day 28 (± 3)	Pre-transplantation levels were inversely associated with grade II–IV aGvHD (*p* = 0.04) Patients with levels below the median had higher CI than patients with levels above the median (46% vs 21%, *p* < 0.01) No significant association was observed between post-transplant Vit E levels and grade II-IV aGVHD No clear association with cGvHD was found	No association with non-relapse mortality	-	One patient (1%) had a pre-transplantation Vit E level below the reference interval for adults (12–42 µmol/L)	Significant increase in post-transplantation plasma Vit E concentration when compared to pre-transplantation plasma Vit E levels (mean difference 6.4 μmol/L, *p* < 0.01)
Carr et al. (2020), NZL, PCS [31]	*n* = 38 (58% male) Age: 57 (8) Allo: 14% Auto: 86%	Not described	Vit C ***(N)***	-	Blood samples were collected prior to SCT, at 1 week, 2 weeks and 4 weeks following SCT	-	-	-	Pre-transplantation 8% had Vit C deficiency (i.e., < 11 µmol/L). Day 14, 34% had Vit C deficiency Day 28, 10% with Vit C deficiency	The lowest mean Vit C values corresponded with the highest mean C-reactive protein values and lowest mean neutrophil counts (*p* < 0.001), by which time all but one of the participants had febrile neutropenia Low Vit C levels were also associated with elevated oxidative stress
Nannya et al. (2014), JPN, PCS [32]	*n* = 15 (60% male) Age: 48 *** (34–69)* Allo: 100%	Patients who had impaired oral intake received PN w/ multivitamin/ trace elements Formulation and duration not described	Vit B1, Vit B6, Vit B9, Vit C, Vit K, Vit E, ***(Y)***	-	Blood samples were measured before SCT, Days 0, + 7, 14, 21 and 28	-	-	-	Average Vit C was under normal range (5.5–16.8 μg/mL) from Day 0 to Day 28 93% cohort at Day 0, 100% at Day 7, 77% and 69% for Days 21 and 28 had Vit B1 values below the lower normal limit (20–50 ng/mL) 50% of cohort had folate levels below the lower normal limit pre SCT and on Day 0. 21% cohort at Day 14 had folate values below the normal range (normal > 3.1 ng/mL) Vit E levels were within the normal range (0.75–1.41 mg/dL) for everyone throughout the acute phase Vit K levels increased up to 10 times the normal upper limit (0.15–1.25 ng/mL) at Day 14 and 21	Vit C deficiency was significantly associated with C-reactive protein and ferritin (inflammatory markers) Vit K overload associated with administration of parenteral supplementation
Rasheed et al. (2019), USA, PCS [33]	*n* = 15 (40% male) Age: 50 (31–65)* Allo: 73% Auto: 27%	Not described	Vit C ***(N)***	-	Blood samples drawn prior to transplant and Days 0, + 14, 30, 60 post SCT	26.7% of cohort had aGVHD and 26.7% had cGVHD. Association not studied due to relatively low event rate	-	No significant association between severity of mucositis and Vit C level	40% of cohort were deficient (< 30 μmol/L) at baseline Day 0, 66% deficient Day 14, 86% deficient Day 30, 50% deficient Day 60, 42% deficient	Significantly lowered Vit C levels on Day 0 (40.8 vs 27.3 μmol/L, *p* = 0.03) and Day 14 (40.8 vs 21.5 μmol/L, *p* = 0.003) than baseline and continued low levels to Day 60
Eicher et al. (2020), CHE, PCS [34]	*n* = 183 (70% male) Normal VitD: 61 (24–74)* Low VitD: 60 (25–77) Auto: 100%	Not described	Vit D ***(N)***	-	25-OH-Vit D on the day of hospital adm before SCT	-	Vitamin D level > 52 nmol/L was associated with higher PFS (19.3 vs 14.3 months, *p* = 0.019) and OS (21.3 vs 17.1 months, *p* = 0.011) and lower overall mortality (14% vs 28%, p = 0.019) and 2 years mortality (10% vs 25%, *p* = 0.008)	-	56% of cohort had Vit D level ≤ 52 nmol/L at baseline. Vit D level < 50 nmol/L is defined as deficiency. Vit D level between 52.5 and 72.5 is insufficient. Vit D levels of all participants were under 72.5 nmol/L	-
Urbain et al. (2012), DEU, PCS [35]	*n* = 102 (63% male) 56 (11) Allo: 100%	22.5% (*n* = 23) of cohort received PN (including 200 IU vit D) on > 20% of the days (range, 21–84% of the days)	Vit D ***(Y)***	8 patients took daily supplements at baseline [median 200 IU daily (range 100–500)], only 1 patient did so on Day 100 23 patients had PN (included 200 IU vit D) on > 20% of the days (between baseline to Day 30)	Blood samples were collected on baseline, Days 30 and 100 post SCT	43% of cohort had grade 2–4 aGvHD No significant association was observed between low Vit D level and incidence of aGvHD	-	-	89.2% of cohort had Vit D level under the normal range (30–70 ng/ml) at baseline, and 23.5% had extremely low Vit D level (< 10 ng/ml). The mean serum levels were 15.5 ± 8.7 ng/ml + 30 and 14.9 ± 7.5 ng/ml + 100	Only higher body fat mass remained an independent risk factor for reduced baseline concentrations (*p* = 0.007)
Raoufinejad et al. (2019), IRN, RCT [36]	*n* = 80 (67.5% male) Age: VitD:47.5 (13.5) CON: 46.9 (14.4) Auto: 100%	3 patients received PN. (range, 3–8 days) Formulation not described Patients who were unable to take oral calcitriol were excluded	Vit D ***(Y)***	Oral capsules of calcitriol 0.25 μg or placebo were administered 3 times daily from Day 0 to Day 30	Serum level of 1,25-OH-Vit D were obtained on baseline, Day 15 and 30 post SCT	-	Two-year RFS was significantly higher in the calcitriol group than the placebo group (77% vs 59%, *p* = 0.03) Calcitriol supplement had no significant association with OS	No significant association between OM indices and intake of oral calcitriol	-	ALC recovery was significant shorter in the calcitriol group (13 vs 20 days, *p* < 0.001)

*PCS*, prospective cohort study; *RCT*, randomised controlled trial; *RCS*, retrospective cohort study; *PN*, parenteral nutrition; *EER*, estimated energy requirement; *SCT*, stem cell transplantation; *aGVHD*, acute Graft versus host disease; *cGVHD*, chronic Graft versus host disease; *OMI*, oral mucositis index; *MTX*, methotrexate; *CI*, cumulative incidence; *PFS*, progression free survival; *OS*, overall survival; *RFS*, relapse free survival; *adm*, admission; *ALC*, absolute lymphocyte count; *CON*, control; *Mod-PN*, modified Parenteral nutrition solution; *RR range; relative risk; ** SE; *** Median

Out of the eleven studies, eight monitored vitamin levels in the acute post-transplant phase [26, 29–36] and seven documented vitamin levels from pre- to post-transplant [26, 30–36]. Seven studies provided vitamin supplementation during the study period [26–29, 32, 35, 36]. Supplementation encompassed oral multivitamins as well as PN formulations specifically containing the vitamin of interest or intravenous multivitamin supplement administration. Ten studies provided plasma measurements of the studied vitamin (Table 1) with one study dividing its study cohort according to folic acid intake [27].

Definition of the timing of baseline vitamin measurements varied between studies. Seven studies recorded baseline measurements at hospital admission prior to intensive chemotherapy [26, 29, 32–36]. Whereas one had blood samples drawn one day prior to transplant [31], one recorded baseline at Day -23 (± 15 days) [30] and one had plasma vitamin D cryopreserved 1–3 months before transplantation [28].

### Vitamin A

Only one study, High et al. [26], reported vitamin A deficiency and associations with mortality and OM. All patients were supplemented with daily a multivitamin (of prenatal-vitamin strength, amount not specified) either orally or parenterally. Though the timeframe wasn’t specified, 68% of the patient group had at least one hyporetinolemic measurement (≤ 1.05 mol/L) and 31% had at least one plasma concentration below the World Health Organization definition of retinol deficiency (≤ 0.70 mol/L) during the study period. From multivariate analysis, plasma retinol levels were not associated with overall mortality. A significant decline in plasma concentrations was reported at Day 7 post SCT, with the severity of mucositis reported as a strong predictor (*p* = 0.001). However, values recovered to baseline concentrations by Day 14 [26]. The certainty of evidence for the association of vitamin A deficiency with mortality and OM was rated as very low according to the GRADE assessment, downgraded due to very serious risk of bias and serious imprecision (Table 2).

**Table 2:** GRADE_a_ certainty of evidence of patient outcomes in 11 included studies of the vitamin requirements of patient undergoing SCT

	Certainty assessment						
No. of studies	Study design	Risk of bias	Inconsistency	Indirectness	Imprecision	Publication bias	Certainty
Vitamin A							
Mortality [26]							
1	Observational	Very serious^b^	Not serious	Not serious	Serious^c^	Not serious	*Very Low* ⨁**◯◯◯**
OM [26]							
1	Observational	Very serious^b^	Not serious	Not serious	Serious^c^	Not serious	*Very Low* ⨁**◯◯◯**
Vitamin E							
GvHDe [30]							
1	Observational	Very serious^b^	Not serious	Not serious	Serious^c^	Not serious	*Very Low* ⨁**◯◯◯**
Mortality [26, 30]							
2	Observational	Very serious^b^	Not serious	Not serious	Serious^c^	Not serious	*Very Low* ⨁**◯◯◯**
Folate (Vitamin B_9_)							
GvHD [27]							
1	Observational	Very serious^b^	Not serious	Not serious	Serious^c^	Not serious	*Very Low* ⨁**◯◯◯**
Mortality [27]							
1	Observational	Very serious^b^	Not serious	Not serious	Serious^c^	Not serious	*Very Low* ⨁**◯◯◯**
OM [27]							
1	Observational	Very serious^b^	Not serious	Not serious	Serious^c^	Not serious	*Very Low* ⨁**◯◯◯**
Vitamin D							
GvHD [28, 35]							
2	Observational	Very serious^b^	Serious	Not serious	Serious^c^	Not serious	*Very Low* ⨁**◯◯◯**
Mortality [28, 34, 36]							
3	Observational, 1 RCT	Very serious^b^	Serious	Not serious	Serious^c^	Not serious	*Very Low* ⨁**◯◯◯**
OM [36]							
1	RCT	Not serious	Not serious	Not serious	Very serious^f^	Not serious	*Low* ⨁⨁**◯◯**
Vitamin C							
GvHD [33]							
1	Observational	Very serious^b^	Not serious	Not serious	Very serious^f^	Not serious	*Very Low* ⨁**◯◯◯**
OM [33]							
1	Observational	Very serious^b^	Not serious	Not serious	Very serious^f^	Not serious	*Very Low* ⨁**◯◯◯**

^a^GRADE = Grading of Recommendations Assessment, Development, and Evaluation

^b^Risk of bias was “very serious” due to the included studies employing an observational study design

^c^Imprecision was “serious” due to the small sample size (> 400) of the included studies

^d^OM = Oral Mucositis

^e^GvHD = Graft-versus-Host-Disease

^f^Imprecision was “very serious” due to the small sample size (> 100) of the included studies

### Vitamin B1 (Thiamine)

Nannya et al. [32] was the only study that monitored vitamin B1 status. The study noted that 92% of patients had either adequate oral intake or parenteral multivitamin formulation with no specification on the duration or proportion of each. Serum vitamin levels were monitored throughout the first month post-transplant with mean levels in the normal lower limit at hospital admission (20 ng/mL [SD 4.9]), Day 21 (25 ng/mL [SD 15]) and Day 28 (28 ng/mL [SD 18]), whilst deficient at Day 0 (17.4 ng/mL [SD 4.5]) and Day 7 (17.8 ng/mL [SD 3.1]) post SCT. Normal range was 20–50 ng/ml. Statistical analysis from baseline were not reported [32].

### Folate (folic acid)

Two studies provided post-transplant folic acid supplementation (not including studies using folinic acid), where one monitored serum folate levels, and one assessed if there was any association with clinical outcomes [27, 32]. Both studies did not specify how many patients received supplements nor the duration of supplementation. Robien et al. [27] stratified the study cohort according to folic acid intake levels from a calculated daily average in Days 0–18 post-transplant and determined the association of these levels with the development of aGvHD, OM and overall mortality. They reported a non-significant association with a multivariate analysis, for risk of aGvHD, relapse and survival for those consuming above and below the US recommended dietary intake of folic acid, with no clear associations between average OM Index scores and higher folic acid intake. Nannya et al. [32] monitored vitamin levels and only reported on those who were deficient. Fifty percent of participants had deficient vitamin levels (< 3.1 ng/mL) at baseline and Day 0 (2.9 ng/mL), though the prevalence of folate deficiency dropped to 21% at Day 14 when most patients had commenced PN [32]. The certainty of evidence for aGVHD, OM and overall mortality was rated as very low according to the GRADE assessment, downgraded due to very serious risk of bias and serious imprecision (Table 2).

### Vitamin C

Four studies monitored vitamin C levels [29, 31–33] with two studies providing supplementation to participants, though the timeframe was not specified [29, 32]. Three studies [31–33] recorded levels where patients were deficient both pre- and post-transplant (Table 1). The two studies which only monitored ascorbic acid levels [31, 33], found significant decreases in vitamin levels at Day 14 post-transplant compared to baseline (*p* < 0.01 and *p* = 0.003 respectively). Nannya et al. [32], recorded average levels to be lower normal range at baseline with all other measurements at a marked deficiency until the conclusion of the study at Day 28. Statistical analysis was not utilised when comparing to baseline values in this study with data interpreted from displayed graphs and no detail provided on the proportion of patients on PN. Jonas et al. [29] provided 700 mg vitamin C daily with vitamin levels significantly increasing over time in the cohort with all values within normal range.

Only one study measured the association between development of oral mucositis with vitamin C levels. Rasheed et al. [33] reported that patients with Grade ≤ 1 mucositis had a non-significant higher serum ascorbic acid on Day 14 (29.6 ± 19.3 µmol/L) than those with mucositis Grade ≥ 2 (16.9 ± 7.7; *p* = 0.1). The association between ascorbic acid levels and GvHD could not be assessed due to relatively low event rate with a small cohort. The certainty of evidence relating vitamin C status was rated as very low for GvHD and OM (Table 2).

### Vitamin D

Four studies measured vitamin D levels [28, 34–36]  with Urbain et al [35] noting daily supplementation to Day 100 for some patients, Glotzbecker et al. [28] supplemented the study population though the frequency and duration was not specified, Raoufinejad et al. [36] administered calcitriol 0.25 μg capsules thrice daily from transplantation to Day 30 and Eicher et al. [34] monitored values pre-transplant with no post SCT monitoring or supplementation. Glotzbecker et al. [28] divided the cohort into vitamin D levels above and below 25 ng/mL based on serum samples at baseline with all patients who were supplementing were in the > 25 ng/mL cohort. Urbain et al. [35] measured serum 25(OH)D concentrations to identify factors, which included daily supplementation, that impacted levels from early transplant to 100 days post-transplant. All studies that reported on deficiency [28, 34, 35] showed high levels of insufficiency/deficiency, though the definition of deficiency varied across papers. At hospital admission, Urbain et al. [35] reported 89.2% had levels below normal range (< 30 ng/ml) with 23.5% of those deficient (< 10 ng/ml). Deficiency was not recorded at Day 100 though a non-significant 8.4% decrease was noted since admission (from 16.4 ng/ml SD 8.9 to 14.9 ng/ml SD 7.5) [35]. Sixty percent of patients in Glotzbecker et al. [28] were measured as deficient (< 25 ng/mL) at baseline and all participants in Eicher et al. [34] recorded insufficient levels of vitamin D at baseline (< 72.5 nmol/L).

Two studies reported on the association of GvHD development with serum vitamin D levels [28, 35]. Both studies reported no significance in Grades II-IV aGvHD at Day 100 post-transplant, though Glotzbecker et al. [28] reported that in a multivariable competing risk model low pretransplant vitamin D level remained a significant independent factor associated with the development of cGVHD (HR = 5.26, OR = 1.3–20.0, *p* = 0.02). Three studies [28, 34, 36] assessed mortality/survival. Two year mortality was lower in the normal vitamin D group as compared to the low vitamin D group at hospital admission (14% vs 28%; *p* = 0.0191) in Eicher et al. [34] whereas non-significant differences were noted in 3-year progression free survival in Glotzbecker et al. [28] and overall survival in Glotzbecker et al. [28] and Raoufinejad et al. [36]. Though Raoufinejad et al. [36] did identify a higher two-year relapse-free survival in those supplementing with calcitriol capsules compared to placebo (77.0%, SE = 7.0% vs. 59.0%, SE = 8.0%; *p* = 0.03).

Only one study measured OM association with vitamin D status with Raoufinejad et al. [36] reporting no significance with OM incidence between those taking calcitriol capsules and the placebo group. The GRADE certainty of evidence relating to mortality and GvHD to vitamin D status was rated as very low as it was downgraded due to very serious risk of bias and serious imprecision whereas it was rated low for OM due to serious imprecision (Table 2).

### Vitamin E

Four studies [26, 29, 30, 32] monitored tocopherol levels with three recording supplementation use [26, 29, 32]. Gjaerde et al. [30] was considered nil supplementation as PN and supplement use and formulations were not described. All studies reported that α-tocopherol levels were within normal range in the acute phase post-transplant. The studies which provided supplementation showed decreases in plasma vitamin levels in the acute stage post-transplant (Day 7–14) when compared to baseline though none were deficient. Gjaerde et al. [30] reported a significant increase in serum levels at follow-up (Day 28 ± 3) (mean difference 6.4 μmol/L, CI: 3.1–9.7 μmol/L; *p* < 0.01) and only had one patient with a pre-transplantation vitamin E level below the reference range (12–42 µmol/L).

Two studies [26, 30] assessed the association between clinical outcomes and vitamin E levels. High et al. [26] reported no association between overall mortality and vitamin E levels with Gjaerde et al. [30] additionally documenting no clear association with relapse. Only Gjaerde et al. [30] determined an association between vitamin E levels and development of GvHD. They reported that higher pre-transplantation level (Day –23 ± 15 days) was associated with less aGvHD (*n* = 38, *p* = 0.015). This inverse association with Grades II–IV aGvHD was evident after adjustment for known prognostic factors for aGvHD (HR 0.68 per 10 µmol/L increase, [CI]: 0.47–0.98). No clear association was found with cGvHD (95% CI 0.74–1.38). The certainty of evidence for mortality and GvHD in relation to vitamin E status was rated as very low according to GRADE assessment due to the fact it was downgraded due to very serious risk of bias and serious imprecision (Table 2).

## Discussion

This is the first systematic review to investigate vitamin requirements throughout the acute transplant phase for adult patients undergoing SCT. The body of literature investigating vitamin levels and the association with post SCT outcomes is limited. Of the 11 studies included in this review, eight studies monitored vitamin levels within the first 100 days post-transplant and only four of these studies monitored vitamin levels without supplementation. This review found that the relationship between vitamin status and risk of post-transplant OM, GvHD and mortality remains uncertain, with the GRADE certainty of evidence being very low or low across these outcomes. It is unclear whether patients undergoing SCT are at higher risk of vitamin B1, vitamin C, vitamin D, vitamin B9 (folate), vitamin A and vitamin E deficiency. However, all four studies that investigated vitamin D status during SCT identified levels of deficiency or insufficiency prior to transplant [28, 34–36].

### Water-soluble vitamins

As vitamin B1 (Thiamine) requirements are heightened during periods of increased metabolic activity, patients undergoing SCT may have increased vitamin B1 needs due to a likely hypermetabolic state [17]. Only one study included in this review monitored vitamin B1 [32], and found that SCT recipients may be at risk of deficiency, despite being supplemented with a multivitamin. As there have been minimal publications supporting the association between SCT and vitamin B1 deficiency, the justification for routine vitamin B1 supplementation is limited.

Only one study investigated folate status during SCT [32] and found that SCT recipients may be susceptible to folate deficiency, supporting previous findings made by Link et al. [37]. It should be noted that the observational study by Link et al. [37] was not included in this review due to their inclusion of paediatric participants. Although these findings suggest that this population may be at risk of folate deficiency and that supplementation may be indicated, the overall strength of evidence is weak and recommendations surrounding supplementation cannot be made. Robien et al. [27] found no association between folic acid intake and adverse clinical outcomes, and although this study had the largest sample size included in this review (n = 311), the certainty of evidence was very low. Future research is needed to clarify folate requirements throughout SCT.

Patients with haematological cancers have been shown to have reduced serum vitamin C levels [38–40]. Systematic inflammation caused by the conditioning therapy may impact vitamin C status [41, 42]. Several studies included in this current review found a high incidence of deficiency [31–33], which aligns with vitamin C concentrations observed in critically ill patients [43, 44]. The inverse correlation between vitamin C and C-reactive protein noted in one of the studies [32] reviewed, highlights the difficulty of assessing vitamin status in an inflammatory state. An increase in oxidative stress may be both a contributing factor and a consequence of low vitamin C levels [45]. As such, interpretation of these findings must consider the impact inflammatory markers have on vitamin C and other antioxidant levels. Low vitamin C levels may not indicate deficiency but rather a state of inflammation [46], highlighting the difficulty in monitoring vitamin levels and assessing requirements in patients undergoing SCT. The association between vitamin C levels and outcomes OM and GvHD and remains uncertain due to very serious risk of bias and imprecision.

### Fat-soluble Vitamins

Paediatric studies have found that patients are at risk of vitamin A deficiency during SCT [47, 48]. However, it is uncertain if vitamin A status during SCT is associated with mortality and risk of OM, with only 1 study in this current review investigating vitamin A levels and clinical outcomes [26]. Despite the severity of OM being associated with low plasma retinol levels post-transplant [26], the certainty of evidence was rated as very low, downgraded due to the very serious risk of bias associated with the observational study design and small sample size. As the evidence is uncertain for the adult population, further research is needed to investigate vitamin A requirements during SCT to help clarify requirements and inform clinical practice.

Our findings show high prevalence of vitamin D deficiency/insufficiency prior to transplantation [28, 34, 35], supporting previous findings from meta-analyses in cancer populations [49, 50] and studies with paediatric SCT recipients [51, 52]. Prolonged hospital stays which limit sun exposure, reduced intake and potential malabsorption may be contributing factors [3, 51]. Vitamin D deficiency is a concern for critically ill patients due to the important role it plays in immune function [53]. Furthermore, for SCT recipients, vitamin D demands may be increased due to it’s role in hematopoiesis [54, 55]. Although vitamin D has the potential to reduce the risk of infections [53], this current review found no benefit in supplementing vitamin D to reduce the incidence, severity or duration of OM [36]. There were inconsistent findings reported in the two studies that investigated the association between vitamin D levels and risk of GvHD and overall survival [28, 35]. However, previous findings from Caballero-Velazquez et al. [52], which included both adult and paediatric participants, indicate that vitamin D supplementation can reduce the incidence and severity of cGvHD. It is important to note that studies focusing only on GvHD were excluded, therefore this current review did not include all studies that investigated the relationship between vitamin D levels and GvHD. Factors that may explain the inconsistencies in findings include; different vitamin D deficiency cut offs, < 25 ng/mL (equivalent to 62.5 nmol/L) [28] versus < 50 nmol/L [34] and different measurement tools. However, Glotzbecker et al. [28] corroborates with results from other published studies that found low vitamin D levels are a risk factor for aGvHD in patients undergoing organ transplant [56–58]. From this current review, it is uncertain whether vitamin D status has an impact on overall survival. However, SCT survivors often have prolonged vitamin D deficiency and poor bone health in the months and years following transplant [59–61]. Based on these findings, monitoring of vitamin D levels prior to and post transplantation should be considered, with supplementation where appropriate.

All four studies [28–30, 32] that monitored vitamin E levels found no association between SCT and vitamin E deficiency. However, there was some inconsistency with the prevalence of vitamin E deficiency reported. This difference may be attributed to differing parenteral formulas. Participants in Gjaerde et al. [30] received a parenteral formula rich in fat-soluble vitamins, including vitamin E, although the specific formulations were not described. No association between overall mortality and α-tocopherol levels were reported [26, 30], although Gjaerde et al. [30] did find a significant correlation between pre-transplantation levels and aGvHD. Whilst the mechanism is unclear, vitamin E could reduce the endogenous release of reactive oxygen species [62], which may contribute to GvHD in the gastrointestinal tract [63]. However, due to the relatively small sample size (*n* = 115) and observational study designs, the certainty of the evidence is very low.

Only one study monitored vitamin K status during SCT and observed a significant excess associated with the commencement of PN although the amount provided was unclear [32]. However, due to the rarity of toxicity and the adverse consequences of deficiency, especially in patients receiving prolonged antibiotic therapy [64], removal of vitamin K from PN during SCT may not be clinically appropriate.

### Limitations

The findings from this current systematic review must take into consideration the possible limitations of the studies included. Firstly, measurement of plasma micronutrient levels in the presence of systematic inflammation may be unreliable and results may not be representative of true nutritional status [46]. Therefore, interpretation and accuracy of vitamin serum levels and nutrition biomarkers taken from critically ill patients must be considered. As SCT patients often see fluctuations of their blood serum levels, this may not be indicative of their current nutritional status [65]. Only two of the studies reported on inflammatory markers that impact serum vitamin levels, such as C-reactive protein. Comparison between studies was difficult due to variations in the definitions of deficiency, differences in the diagnoses of OM or GvHD, limited reporting at timepoints and/or lack of statistical analysis reported. Most of the studies did not record oral intake or did not clearly specify the formulas used for nutrition support therapy [26, 28, 30–34, 36], meaning analysis of these confounding factors could not be completed. Of the seven studies that reported the use of PN support, only three stated the duration of the supplementation period and only one of these studies commented on the adherence.

Overall, a limited number of studies were found in this review, with the weight of the literature focusing on the efficacy of EN or PN protocols [43, 66] rather than investigating the micronutrient requirements of this patient group. Additionally, due to the exclusion of studies focusing on a GvHD population, this review did not capture data that may have highlighted the differing nutritional needs of this group. In particular, the prevalence of Vitamin D deficiency observed in previous research [67]. Therefore, the importance of Vitamin D in immune function following SCT [68] may be underrepresented in this review. As this review had a specific focus on vitamins and not vitamin derivatives, it may have excluded papers showing a possible link between supplementing varying derivatives of vitamins and improved clinical outcome, for example, Folinic Acid [69, 70]. Lastly, few studies examined the adult population specifically, with the majority of existing research in this area being within the paediatric population or including a mix of adults and children [44]. This review has identified further opportunities for future research, such as the evaluation of trace element/ mineral requirements during SCT and micronutrient requirements during chronic GvHD. 

## Conclusion

Vitamin adequacy for prevention of adverse clinical outcomes has become an area of focus in clinical practice and research. In general, there are limited well-designed and sufficiently powered studies assessing such parameters in adult patients undergoing SCT. This review highlights the gap in current knowledge relating to vitamin requirements and the need for supplementation during SCT. It remains uncertain whether vitamin levels impact the risk of aGvHD, OM, or mortality. Additionally, it is unclear if SCT increases the demand for certain vitamins and thus increases the risk of deficiency, though monitoring vitamin D levels prior to and post transplant should be considered. In the absence of high-level evidence to guide practice in this area, clinicians need to continue using clinical judgement to provide supplementation where appropriate. If oral intake or nutrition support is suboptimal resulting in a decreased intake of micronutrients, a multivitamin supplement may be indicated. Additionally, EN and PN formulations should be selected carefully to ensure appropriate vitamin and mineral provision. Further large observational studies or randomised control trials are required to investigate the potential influence of vitamin levels on clinical outcomes. This will aid in improving patient-centered care and establishing nutritional protocols guiding supplementation.
